# Blood-letting cupping therapy for incision infection after laparoscopic surgery: a case report

**DOI:** 10.3389/fmed.2026.1875174

**Published:** 2026-07-20

**Authors:** Chao Wang, Yingjun Liu, Haijuan Zhang, Chuanlong Zhou, Lu Li

**Affiliations:** 1 Department of Acupuncture and Moxibustion, The Third Affiliated Hospital of Zhejiang Chinese Medical University (Zhongshan Hospital of Zhejiang Province), Hangzhou, China; 2 Department of Acupuncture and Moxibustion, Pan'an Hospital of Traditional Chinese Medical, Jinhua, China

**Keywords:** blood-letting cupping therapy, case report, cupping, incision infection, laparoscopic surgery

## Abstract

Laparoscopic surgery, as a minimally invasive technique, has gained widespread recognition in clinical practice over recent decades. However, surgical site infection (SSI) following laparoscopy remains a concern that cannot be overlooked. SSI refers to infections related to surgery that occur within 30 days after the operative procedure at or near the surgical incision, or within 12 months if an implant is placed, and includes superficial incisional, deep incisional, and organ/space infections. According to current epidemiological data, the incidence of incisional SSI after laparoscopic surgery ranges from 0.22 to 27.03%. Although the majority of patients recover from this complication, worsening of the infection can lead to prolonged hospitalization, the requirement for reoperation, and even elevated mortality, particularly among immunocompromised patients. This issue remains a key concern for surgeons. Here, we present the clinical course of a 75-year-old female patient who developed incisional surgical site infection after laparoscopic intra-abdominal lesion biopsy for cholangiocarcinoma. In this individual case, the patient manifested typical infectious manifestations including incision inflammation, elevated CRP level, and fever. After receiving a total of five sessions of adjuvant blood-letting cupping therapy tailored to her clinical condition, the local incision infection was gradually resolved, and the patient's inflammatory markers returned to normal range. No infectious recurrence was observed during the 3-month postoperative follow-up.

## Introduction

Laparoscopic surgery has become the preferred approach for many surgical procedures due to its advantages of minimal trauma and rapid recovery (1). However, postoperative incisional surgical site infection (SSI) remains a common and troublesome complication that may negatively affect postoperative recovery (2). The development of laparoscopic incision infection is multifactorial, closely related to surgical operation, perioperative management, and patients' underlying physical status and immune condition. Conventional mainstream management mainly includes standardized wound care, systemic antibiotic administration, and surgical debridement when necessary, yet these routine approaches have inherent limitations for complex superficial wounds. Long-term broad-spectrum antibiotics may disrupt intestinal flora and induce bacterial drug resistance (3). Therefore, exploring safe and minimally invasive complementary therapies is clinically meaningful for optimizing individualized postoperative wound management.

Cupping therapy is a traditional external physical intervention that creates localized negative pressure by evacuating air inside special containers attached to the skin surface, generating mild mechanical stimulation to regulate local qi-blood circulation and inflammatory status for disease treatment (4). Cupping therapy is primarily categorized into dry cupping and wet cupping (also named blood-letting cupping or Hijama). Cupping therapy has a long cross-civilization history. Originating around 1,500 BC in ancient Egypt, it was documented in the Ebers Papyrus and temple murals for pain and fever relief. Hippocrates and Galen adopted it to balance bodily humors in ancient Greece. Early Han Dynasty China developed horn-originated cupping for inflammatory disorders. Named al-hijama, wet cupping became core detox treatment in Arabic-Islamic medicine, and was also widely utilized in Traditional Korean and Unani medicine to manage various inflammatory lesions. As a classic external therapy of traditional Chinese medicine, blood-letting cupping has been widely used for adjuvant management of various inflammatory and soft tissue lesions. Multiple mechanistic and clinical studies confirm that blood-letting cupping stimulates endothelial nitric oxide release to dilate subcutaneous capillaries, accelerates lymphatic clearance of inflammatory exudates, and downregulates pro-inflammatory cytokines such as TNF-α and IL-6. These effects collectively improve local microcirculation, clear inflammatory exudates, modulate regional immune responses, and facilitate superficial postoperative wound repair while lowering local infection risks (5–7).

Despite its clinical benefits, cupping carries potential adverse events if operated improperly. Common mild adverse events include transient local ecchymosis, temporary pruritus, and mild skin erythema; preventable severe adverse events mainly consist of thermal burns related to fire cupping, secondary skin infection, scarring, and vasovagal syncope, most of which stem from non-standardized sterilization, uncontrolled suction pressure or excessively long retention time.

To reduce related risks, unified standardized safety protocols are required for clinical implementation: (1) All cupping containers and puncture tools must undergo high-temperature steam sterilization before each use; (2) Operators strictly avoid direct skin contact with flame during fire cupping to prevent scald injuries; (3) Negative pressure is limited to a safe low-intensity range for patients with impaired glucose metabolism and weakened tissue repair capacity; (4) Disposable sterile pricking needles are used for single use only; (5) Real-time vital sign monitoring is conducted throughout treatment to prevent dizziness or vasovagal syncope (8).

The present study aimed to report one specific clinical case in which a patient developed fever and incisional infection after laparoscopic intra-abdominal lesion biopsy for cholangiocarcinoma. Serial ultrasonography and routine blood examinations were performed to dynamically evaluate the wound condition and inflammatory level. Blood-letting cupping therapy was applied as an adjuvant complementary treatment for local incision infection. The patient achieved complete wound recovery without recurrence during the 3-month follow-up, providing preliminary observational evidence for the potential value of this TCM therapy in individual postoperative SSI management.

## Case presentation

**Figure 1:**
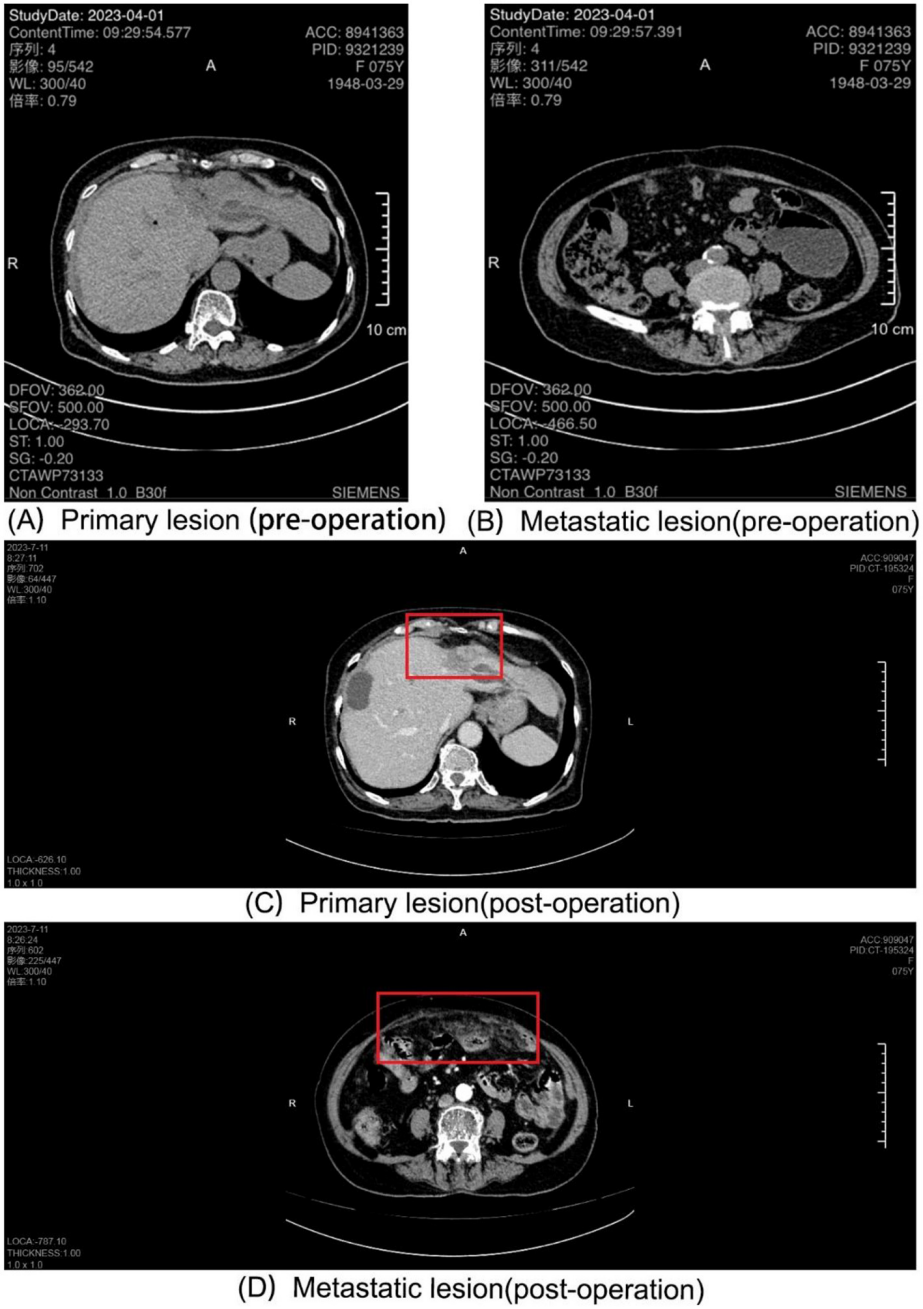
CT imaging of primary and metastatic lesions. **(A)** and **(B)** represent the primary and metastatic lesions on preoperative imaging; **(C)** and **(D)** represent those on postoperative imaging.

On February 14, 2023, the patient presented to Yantai Yuhuangding Hospital with sudden onset of dull pain in the right lower abdomen. Enhanced abdominal CT imaging revealed intrahepatic cholangiocarcinoma (iCCA) with metastasis. On April 1, 2023, contrast-enhanced abdominal CT performed at Sir Run Run Shaw Hospital showed a low-density lesion in the left hepatic lobe, highly suggestive of cholangiocarcinoma, with suspected peritoneal and omental metastasis (Figures 1A, B). Postoperative follow-up CT images of the patient's primary and metastatic lesions are shown (Figures 1C, D). On April 6, 2023, the patient underwent laparoscopic intra-abdominal lesion puncture biopsy at the same hospital. A small curved incision was made below the umbilicus, a pneumoperitoneum needle was inserted into the abdominal cavity, and an ultrasonic scalpel was used to dissect the masses along the edge of omental nodules. The patient maintained stable vital signs after the operation, and the biopsy results confirmed malignant cholangiocarcinoma and peritoneal adenocarcinoma. Postoperative management included analgesia, antibiotics, antiemetics, and acid suppression. The patient also received combined therapy with S-1 capsule 50 mg twice daily plus Atezolizumab for her cancer treatment. The patient was discharged from Sir Run Run Shaw Hospital on April 15, 2023, and was immediately transferred to our acupuncture department on the same day to receive Traditional Chinese Medicine treatment. In addition, the patient had a 10-year history of anxiety and depression, a 20-year history of primary hypertension, and several months of elevated blood glucose without regular hypoglycemic medication, with a glycosylated hemoglobin (HbA1c) level of 7.31%.

**Figure 2:**
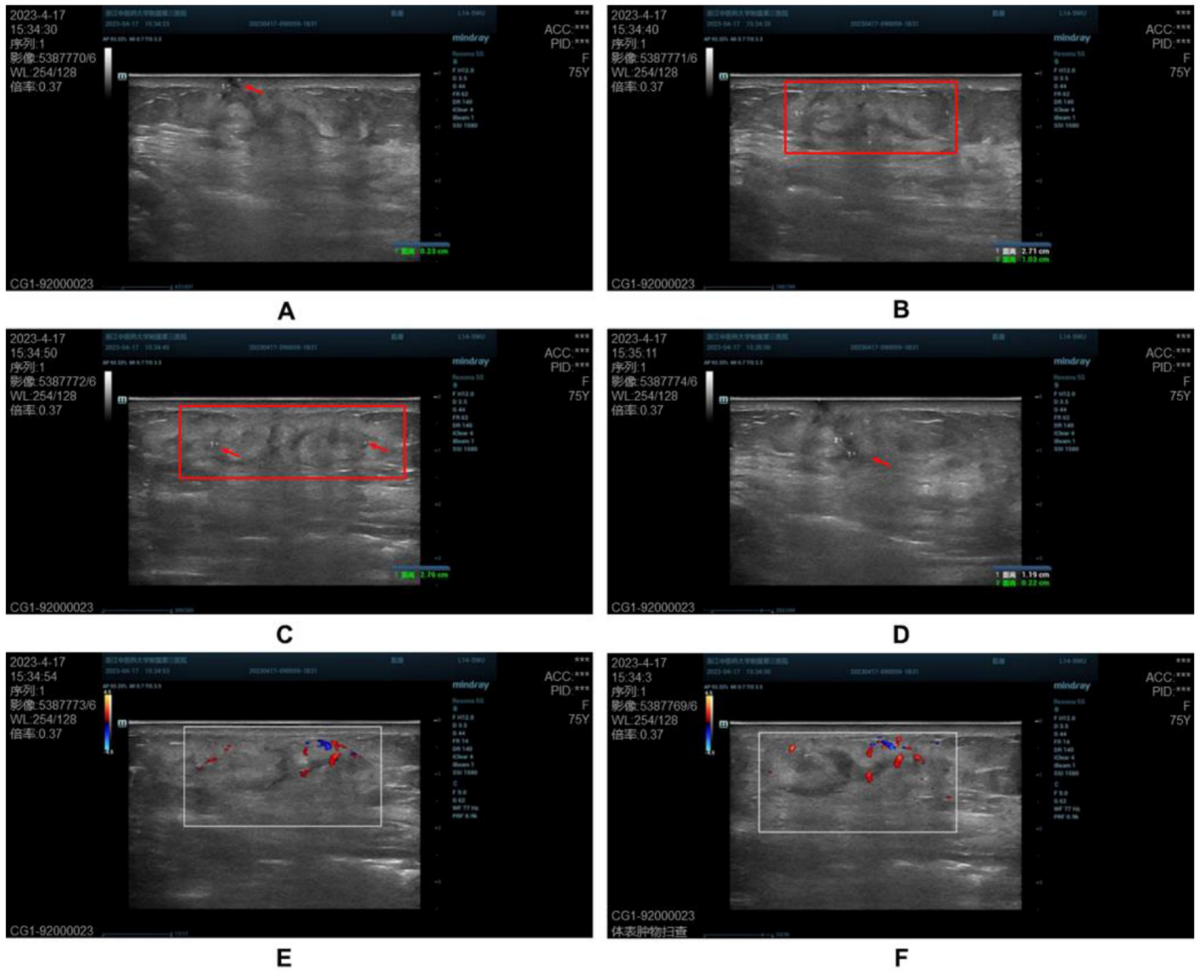
Ultrasonography on the incisional infection site. **(A)** means the tube to the skin; **(B–D)** mean the size of the incisional infection site; **(E and F)** mean the blood flow in the incisional infection site.

Upon admission, the patient presented with a temperature of 39 °C. Antibiotic therapy and antipyretic treatment were administered. On April 17, 2023, ultrasonography was performed on the “mass” in the right abdominal wall, revealing a heterogeneous hypoechoic area measuring approximately 2.71 × 1.03 × 2.76 cm in the subcutaneous layer. Additionally, an oval-shaped erythematous lesion was observed on the skin of the right upper abdominal wall, measuring approximately 2.96 × 0.64 cm, with a slightly elevated local skin temperature. The lesion had poorly defined borders and appeared to contain a narrow hypoechoic to anechoic tract approximately 0.23 cm in width, connected to the body surface. Slightly increased blood flow signals were observed within the lesion (Figures 2E, F).

## Treatment protocol

All blood-letting cupping procedures were conducted by Dr. Chao Wang, a fully licensed TCM physician with standardized training in this technique. Standardized instruments and safety operation specifications were strictly implemented throughout all sessions: ➀ Cupping tools: No.3 standard medical glass cups were adopted; ➁ Negative pressure generation: fire cupping method was used. Given the patient's weakened wound repair capacity caused by abnormal glucose metabolism, low-to-moderate negative pressure was consistently applied to avoid excessive skin traction; ➂ Anti-scald measures: the flame inside the cup was fully extinguished before fitting the cup to the skin, and the cup rim was rapidly cooled to prevent thermal injury; ➃ Disinfection standard: all glass cups underwent high-temperature steam autoclave sterilization before each treatment session. During the whole set of puncture and cupping procedures, the patient reported no stabbing or burning pain; only mild soreness and pulling sensation during cupping retention, which was completely tolerable without additional analgesic intervention.

**Figure 3:**
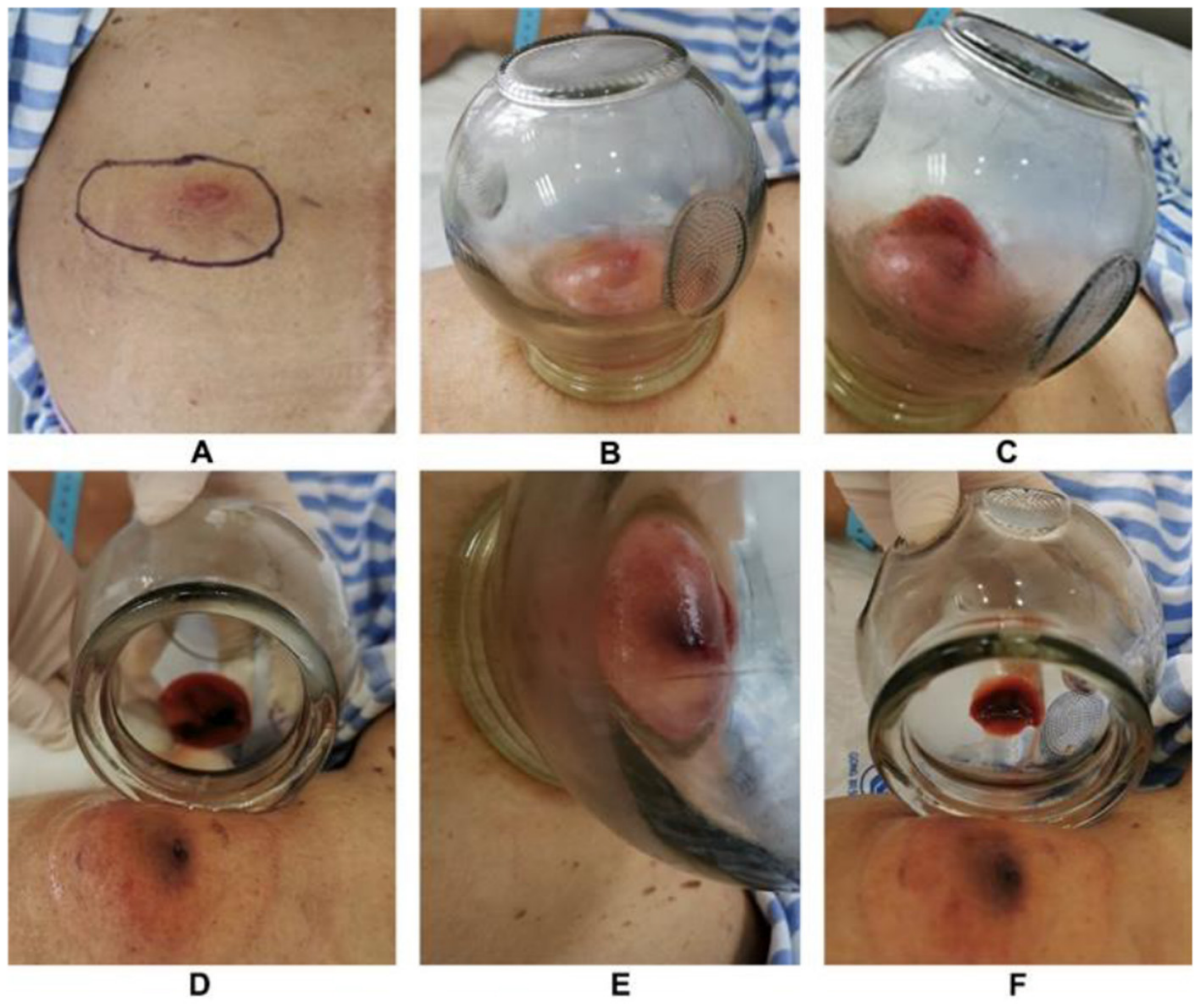
The whole procedure of blood-letting cupping therapy. **(A)** means marking localization; **(B)** means cupping; **(C)** means retaining the cups; **(D)** means drawing out stagnant blood; **(E and F)** mean repeating the cupping procedure again.

The target area was marked and routinely disinfected. Using a disposable sterile syringe needle, rapid puncture was performed multiple times in an area of about 3 × 3 cm, at a depth of approximately 0.5–1 cm, with the center point of infection site as the center. The puncture points were spaced 0.2–0.5 cm apart, with a total of 10–15 punctures. Cupping was immediately applied and the cups retained for 7–8 min. A mixture of blood and pus was successfully drained from the infected site. After suctioning out the stagnant blood, the area was disinfected again, and cupping was repeated. The treatment was administered once every 3–5 days. The first session of blood-letting cupping therapy was performed at 9:00 a.m. on April 21, 2023 (Figure 3). The patient was instructed to lie in the supine position, with the skin of the right upper abdominal wall exposed (Figure 3A). The target area was located and disinfected, followed by needle pricking with a syringe (Figures 3B–D). Cupping was applied twice, each session lasting approximately 7–8 min (Figures 3E, F). Post-procedure disinfection was performed.

### Comparison of subcutaneous lesion scope changes under ultrasound

As is shown in Figure 2, the lesion presented as a localized inflammatory mass with ill-defined borders and heterogeneous echotexture. Multiplanar measurement revealed a maximum longitudinal diameter of approximately 2.71 to 2.76 cm, a transverse diameter ranging from 1.03 to 1.19 cm, and a tube width of 0.23 cm. Color Doppler imaging detected blood flow signals within and around the lesion, indicating active inflammation. No obvious large abscess cavity was identified. These imaging findings were consistent with superficial inflammatory changes following cholecystectomy.

**Figure 4:**
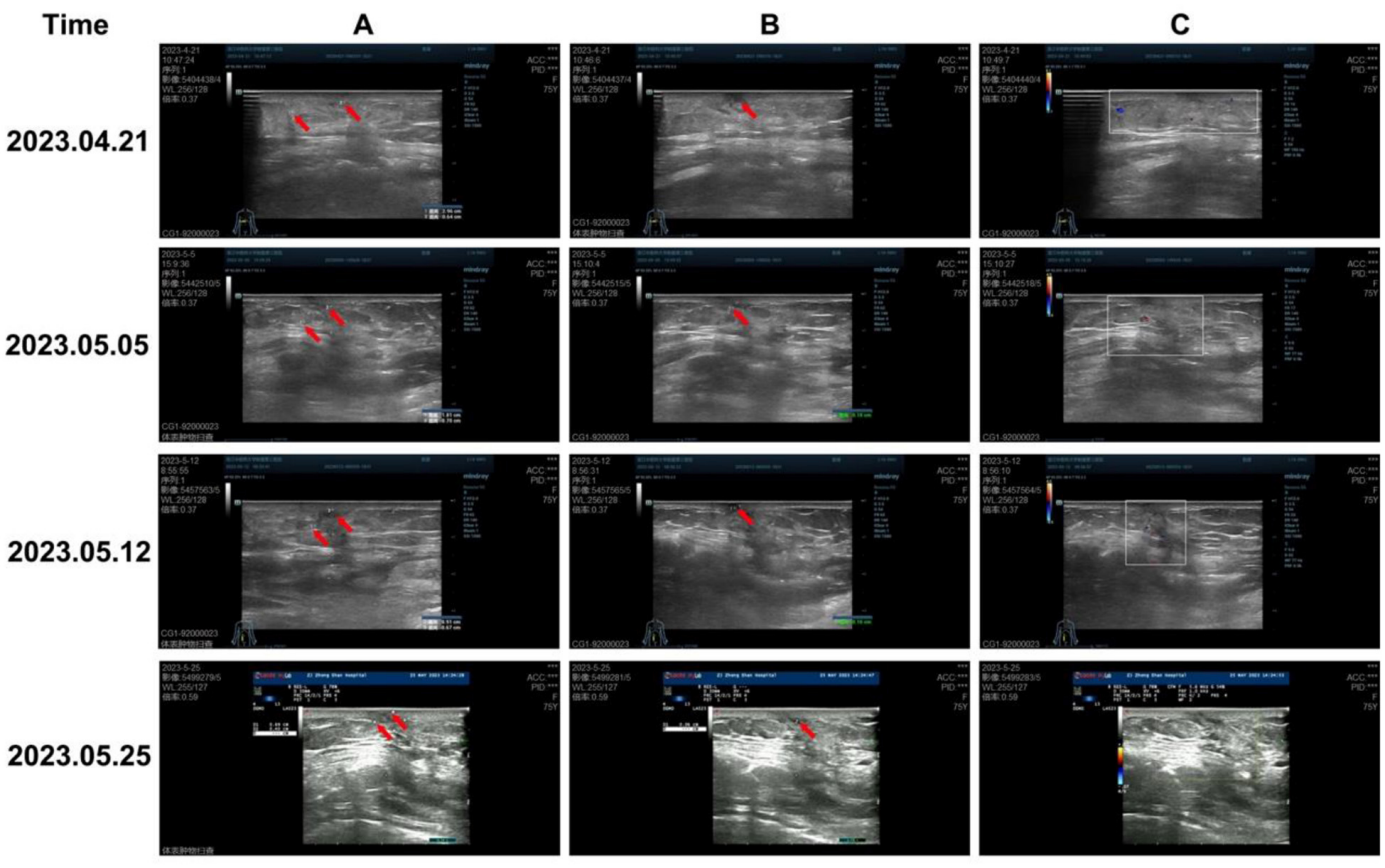
Size changes of incisional infection site on ultrasonography. **(A)** means the length and width of the incisional infection site. **(B)** means the width of tube to skin. **(C)** means the blood flow in the incisional infection site.

**Figure 5:**
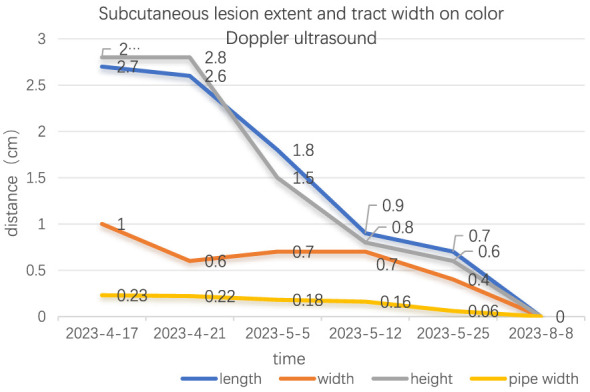
The range of subcutaneous lesions and the change of pipeline width under color Doppler ultrasound.

An immediate post-procedure ultrasound was performed after the first blood-letting cupping therapy at 09:47 a.m. on April 21, 2023 (Figures 4A–C −2023.04.21). The ultrasound indicated that the subcutaneous lesion area was slightly smaller compared to that observed on April 17, 2023 (Figure 2 and Table 1). From April 21, 2023, to May 12, 2023, the treatment was administered once every 3–5 days, totaling 5 sessions during this period. Follow-up ultrasound examinations conducted after each session revealed a progressive reduction in the size of the subcutaneous lesion in the right abdominal wall. Detailed measurements are presented in Figures 4, 5. Notably, after the third treatment, a follow-up ultrasound on May 5, 2023, showed that the tubet-like hypoechoic structure connected to the body surface had become narrower and exhibited discontinuity (Figures 4A–C −2023.05.25).

**Table 1:** The range of subcutaneous lesions and the change of pipeline width under color Doppler ultrasound.

Examination Time	Length	Width	Width of tube
2023-4-17	2.71	1.03	0.23
2023-4-21	2.96	0.64	0.22
2023-5-5	1.81	0.70	0.18
2023-5-12	0.91	0.67	0.16
2023-5-25	0.69	0.40	0.06
2023-8-8	0	0	0

### Changes in inflammatory markers on complete blood count

**Figure 6:**
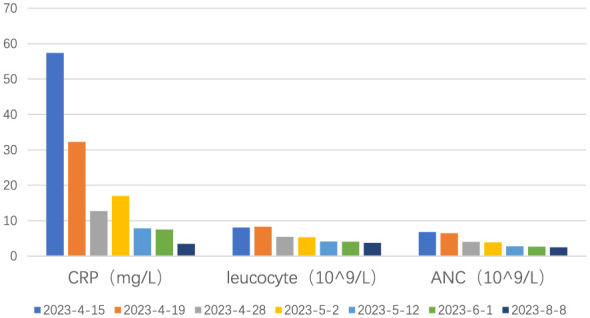
Changes in inflammatory markers on complete blood.

During the treatment and follow-up, the patient underwent multiple routine blood tests. The white blood cell count remained within normal range but showed a downward trend. The neutrophil percentage and absolute count returned to normal levels by April 28, 2023 (Figure 6). C-reactive protein (CRP) returned to the normal range on May 12, 2023. Blood tests indicated no signs of infection in the peripheral blood, accompanied by the absence of fever. Ultrasound of the affected body surface area revealed a significant reduction in the subcutaneous lesion compared to pre-treatment dimensions, with no obvious redness or swelling of the skin at the target site. Additionally, the patient reported no other significant discomfort (Table 2).

**Table 2:** Changes in inflammatory markers on complete blood.

Time points	2023.04.15	2023.04.19	2023.04.28	2023.05.02	2023.05.12	2023.06.01	2023.08.08
CRP (mg/L)	57.34	32.21	12.7	16.97	7.83	7.49	3.45
White cell (10∧9/L)	8.07	8.27	5.42	5.33	4.12	4.06	3.72
Neutrophil percentage (%)	84.0	77.7	73.6	71.8	66.5	65.3	66.3
Absolute neutrophil count (10∧9/L)	6.77	6.43	3.99	3.83	2.74	2.65	2.47

### Skin recovery at the affected site

**Figure 7:**
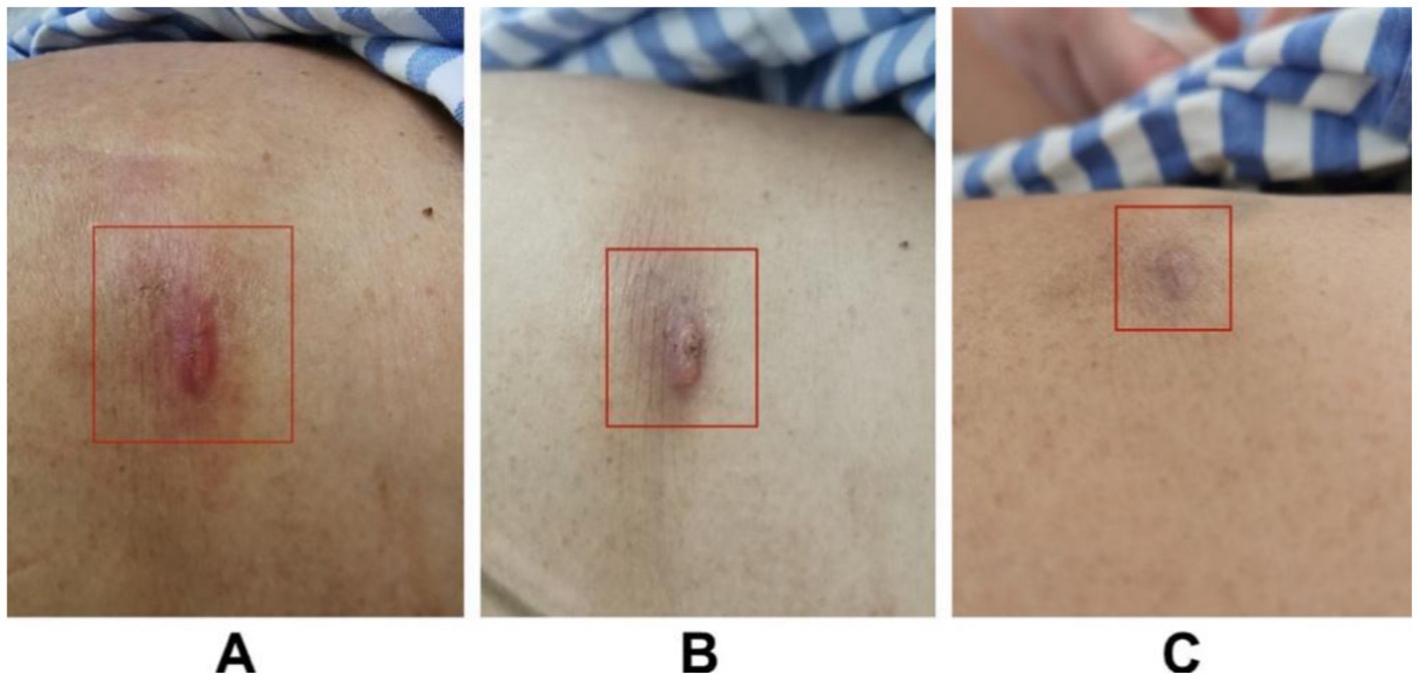
Skin recovery at the affected site on the abdominal wall. **(A)** 20230417 **(B)** 20230504 **(C)** 20230605.

After five sessions of blood-letting and cupping therapy, the erythema and swelling on the skin of the right upper abdominal wall showed significant improvement. The color became lighter, and the area of swelling reduced from 2.96 × 0.64 cm to approximately 0.8 × 0.5 cm, with slight elevation above the skin surface (Figure 7B), indicating remarkable improvement compared to the pre-treatment condition (Figure 7A). During the 3-month follow-ups, the erythema and swelling continued to heal substantially; the skin color returned to nearly normal, and swelling was largely absent. The scar measured approximately 0.5 × 0.3 cm, with no obvious elevation above the skin surface (Figure 7C). As of August 8, 2023, the affected skin area showed no obvious abnormalities, presenting as a well-healed, old elliptical surgical scar.

### Three month follow-up observation

**Figure 8:**
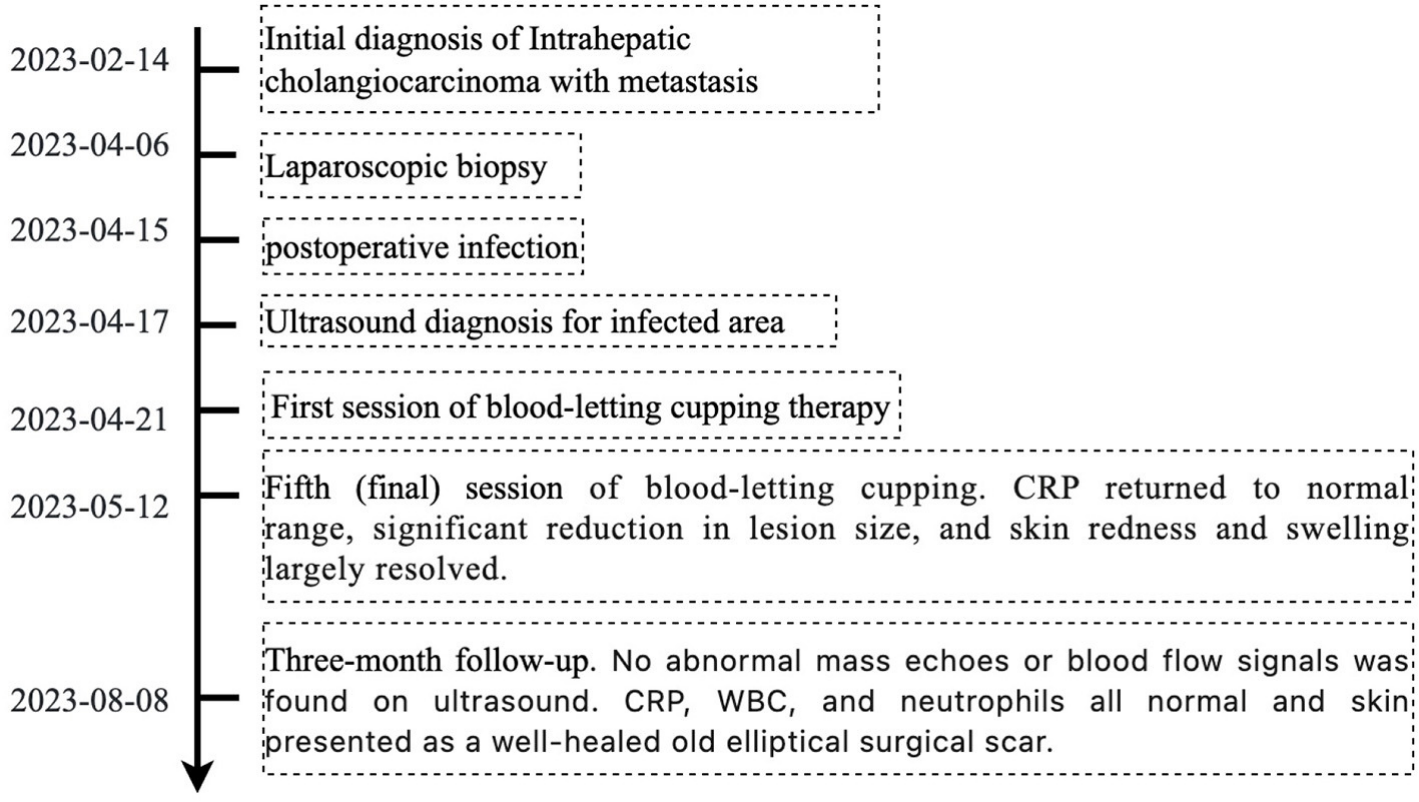
Timeline.

Follow-up color Doppler ultrasound on August 8, 2023 revealed slightly increased echogenicity in the subcutaneous fat layer at the site of the right upper abdominal wall mass, with no significant abnormal mass echoes detected. Color Doppler flow imaging showed no significant abnormal blood flow signals. Concurrent blood tests indicated normal levels of CRP, white blood cells, and neutrophils, suggesting no signs of infection, as detailed in Table 2. The complete timeline of admission, surgery, cupping treatment sessions and follow-up examinations of this patient is summarized in Figure 8.

## Discussion

This case report analyzed the clinical outcome of blood-letting cupping for surgical site infection (SSI) after laparoscopic surgery. Our primary findings indicate that following intervention with blood-letting cupping therapy, the patient exhibited a significant reduction in the extent of the lesion and a marked decrease in inflammatory markers during the healing process of the postoperative incision infection. These outcomes were confirmed through ultrasonographic assessment and inflammatory markers in routine blood tests, proving the effectiveness of the treatment and the ability to help assess the duration of the treatment.

As a classic external therapy of traditional Chinese Medicine, blood-letting cupping is intended to activate blood circulation, remove blood stasis, relieve inflammation, and alleviate pain (9). This procedure involves pinpoint pricking superficial collaterals or lesion areas to induce slight bleeding, followed by cupping suction to eliminate stagnant blood and inflammatory substances (10). Existing clinical evidence indicates that blood-letting and cupping may act as safe adjuvant measure for general postoperative wound complications including incision infection, fat liquefaction and persistent wound exudation, featuring simple operation and rare adverse events. It could assist wound fluid drainage and improve regional inflammatory microcirculation (11, 12).

Multiple mechanistic studies have clarified the biological pathways through which blood-letting cupping may alleviate laparoscopic incision inflammation. Negative pressure stimulates vascular endothelial cells to release nitric oxide, leading to remarkable subcutaneous vasodilation and drastically elevated regional blood flow, which effectively relieves microcirculatory stasis at incision sites induced by intraoperative pneumoperitoneum (5). Quantitative photoacoustic imaging research further verifies that that standardized therapeutic negative pressure dilates subcutaneous capillaries and accelerates lymphatic drainage, rapidly eliminating inflammatory exudates trapped within subcutaneous loose spaces (13). Mechanical stretching generated by cupping activates the heme oxygenase-1 antioxidative and anti-inflammatory pathways, downregulating pro-inflammatory cytokines including TNF-α and IL-6, recruiting macrophages to clear necrotic tissue in wounds, and accelerating local inflammation resolution (14). Laparoscopic incisions are characterized by occluded subcutaneous dead space and accumulated stagnant blood and inflammatory effusion—features that cannot be fully addressed by conventional surgical drainage. Conventional catheter drainage only achieves passive pus evacuation without improving local circulation. In comparison, multi-point superficial pricking during bloodletting cupping unblocks closed subcutaneous gaps, and subsequent cupping suction delivers sustained superficial negative-pressure drainage. The combined intervention may eliminate oxidative inflammatory metabolites and promote angiogenesis and granulation regeneration, producing comprehensive repairing effects that single conventional drainage lacks (6).

The complete wound healing duration of approximately 1 month in this case resulted from the patient's tumor cachexia combined with abnormal glucose metabolism (HbA1c 7.31%), which impaired intrinsic soft tissue regeneration capacity, rather than from limitations inherent to the cupping intervention. The wound was kept open after each session to maintain gentle continuous drainage, which may prevent superficial pseudo-healing with hidden deep abscess cavities and contribute to more stable long-term wound repair compared with closed drainage.

In the setting of laparoscopic surgical incision infections, blood-letting and cupping have synergistic therapeutic effects: bloodletting accelerates local microcirculation and reduces the aggregation of inflammatory cells, while the negative pressure produced by cupping facilitates the clearance of purulent secretions and necrotic tissue. Thus, combined use of these two can effectively control local infectious lesions. Consistent with these mechanisms, our clinical observation showed that, after standardized blood-letting cupping therapy, the patient exhibited continuous shrinkage of the lesion, gradual closure of the peritoneal fistula, and progressive normalization of inflammatory markers including C-reactive protein (CRP) and neutrophil count on color Doppler ultrasound.

Compared with previous reports focusing on uncomplicated postoperative wounds, this case provides preliminary observational data on blood-letting cupping for refractory superficial infection after cholangiocarcinoma minimally invasive surgery in a patient with concurrent mild glucose metabolism disorder and chronic comorbidities. This observation expands the potential applicable population of blood-letting cupping and may offer a minimally invasive complementary option for complex postoperative wounds in cancer patients, enriching the existing clinical evidence base of TCM external therapies. This case merely provides single-case observational experience instead of universal therapeutic conclusions for all surgical site infections.

Standard antibiotics and surgical debridement remain primary standardized treatments for laparoscopic incision infection, with recognized clinical value despite certain limitations such as intestinal flora disturbance and repeated mechanical wound trauma. As a complementary intervention, blood-letting cupping cannot replace standardized antibiotic and debridement regimens which remain the primary management for laparoscopic incision infections. For this individual patient, adjuvant blood-letting cupping may help lower reliance on repeated mechanical debridement and reduce the systemic side effects brought by long-term single antibiotic administration without disrupting routine Western medical treatment. This single-case observation only reflects the auxiliary value of blood-letting cupping in local inflammation control, which may provide a supplementary therapeutic idea for clinicians to optimize individualized wound care plans.

In summary, this case report suggests that adjuvant blood-letting cupping may facilitate wound recovery for SSI after laparoscopic surgery, and early intervention might bring better local anti-inflammatory outcomes. More high-quality clinical trials are required to confirm its general clinical value.

Several limitations of this case report should be acknowledged. First, inconsistent ultrasonic evaluation standards among operators may introduce assessment bias. Second, short-term preoperative antibiotics may act as confounding factors affecting wound recovery. Third, the single-case design cannot support generalized conclusions for all similar patients. Larger-sample standardized clinical trials are warranted to further verify the long-term efficacy and safety of blood-letting cupping for postoperative surgical site infections.

In general, this case illustrates the potential auxiliary value of integrated TCM blood-letting cupping combined with modern surgical management for laparoscopic incision infection, which deserves further clinical exploration.

## Conclusion

After five sessions of blood-letting cupping therapy, the patient with surgical site infection (SSI) following laparoscopic surgery demonstrated favorable resolution of infection and improved wound healing. At the 3-month follow-ups, no recurrence of the incisional infection was observed. All observations are limited to this single case and cannot be extrapolated to all patients with postoperative incision infection. This case only provides preliminary clinical observational evidence; large-scale prospective clinical trials are needed to systematically verify the efficacy of blood-letting cupping as a complementary therapy for SSI.

## Data Availability

The original contributions presented in the study are included in the article/supplementary material, further inquiries can be directed to the corresponding author.
